# The American Fleet at Auckland

**Published:** 1908-12

**Authors:** 


### SELECTION

### The American Fleet at Auckland.

### American Naval Surgeons Entertained by Medical Men of New Zealand

[*New Zealand Medical Journal*, August, 1908]

ON the occasion of the visit of the American Fleet to Auckland, the Auckland Division of the B.M.A. entertained the visiting Naval Surgeons at the Northern Club, on the evening of Friday, August 14, 1908. The entertainment took the form of a supper and smoke concert, to which items were contributed by medical men and leading local amateurs.

Dr. Tracy Inglis, President of the Auckland Division, was in the chair. The meeting was a most representative one, there being present Fleet Surgeon Curtis, and 20 surgeons from the fleet, Staff-Surgeon Glazebrook, H.M.S. Encounter, Surgeon Murphy, H.M.S. Pioneer, about 40 members of the local division, many of whom had traveled long distances in order to be present; Dr. Mason, Dr. Chapple, M.P., and Dr. Martin (Wellington); Dr. Maurice (Greymouth). In addition to members of the medical profession, there were present the American Vice Consul, Hon. J. Mitchelson, Messrs. Gresham (Coroner), Kirker, and Bagnal (Fleet Reception Committee), and H. M. Gore.

His Excellency the Governor, the Premier, the Minister of Public Health, and the Mayor were unavoidably absent as they were being entertained by Admiral Sperry.

The president, in extending a hearty welcome to the guests, said:

*Gentlemen*: I feel it a great privilege that it devolves upon me as President of this Division of the B.M.A. to be able to say a few words of welcome to such an assemblage of medical men, and especially to those of you who come from the great English speaking country, America.

In sending a battleship fleet round the world, the United States has demonstrated not only the will, but the power, to have a large voice in the future settlement of eastern questions. In this matter I am sure all members of the profession hope the results will tend to the preservation of peace; and on such a unique occasion as the present, we have felt it to be a great privilege to welcome to our midst, the medical men attached to the fleet, and we hope that they will take away with them the most pleasant recollections of their stay in New Zealand waters.

In my opinion there is no better training for a naval surgeon than is afforded by the experience of long sea voyages, as they give him opportunities of doing his work under circumstances approximating those of actual warfare.. In a voyage such as the present, you have also the advantage of studying the application of surgical and medical methods under conditions, often difficult, and in constantly varying climates, which must at times test your ingenuity to the utmost. I have no doubt that you, gentlemen, our guests tonight, will, when you once more reach your native shores, be able to add something from the result of your experience to the sum of human knowledge, as far as medicine, and possibly in a greater degree still, surgery are concerned.

There are, gentlemen, quite a number of risks which, as a profession, we run—even on shore—in carrying out our daily routine; but I consider a naval surgeon, in time of war, requires more courage to successfully perform his duty than most of the actual combatants. He has all the risks without the excitement of actual fighting to sustain him in the emergency. Therefore, we quite realise that in the branch of the profession which you have chosen, the value of such an experience as your extended cruise enables you to enjoy, is one which could not be over estimated.

Today the American Schools of Medicine and Surgery are becoming of more and more importance, and are bidding fair to rival the schools of the Old World, as during the past twenty years or so, they have already done in dentistry. The names of the Mayo Brothers, Howard Kelly, Senn and Keen in Surgery, and Osler and Holt in Medicine, are famous throughout the medical world, and, being those of your own countrymen, they must perforce have become household words to you; in this connection I am sure that you will all agree that the rapid advancment which has distinguished the American Schools during the last generation has largely been made possible by the splendid monetary endowments by the rich among your countrymen, who have, perhaps more than any other nationality in the world, furthered the interests of humanity, by increasing the usefulness of the profession to which we all have the honor to belong. I should like also to refer to the splendid work done by your countryman Carroll, in the investigation of yellow fever, which rivals in brilliancy the work of Ross and Bruce in connection with malaria and sleeping sickness. Again we have the brilliant work by the American Naval and Army Staff, who have fought disease in the Canal Zone. By the researches of Gorgas and others, they have made that district habitable, and deserve the highest credit for the wonderfully low mortality from dysentery, malaria, and yellow fever. The work of your Naval and Army Staff in this connection shines out all the more brilliantly, when we recall the ravages of disease amongst those who first attempted the work of forming the Panama Canal.

There is another point in which your countrymen excel, and one which has left the doctors of all nations under an obligation which cannot be over-estimated. I refer to the manner in which your medical books have been produced. The superb illustrations and diagrams in some of your surgical publications are admired and envied by us all, and the adaptation in America of trichromatic printing to the reproduction of technical and scientific illustrations has made the most complex problems very much easier to understand.

I am sure, Gentlemen, that your cruise must have been most instructive, and though, of necessity, you may have had to bear the burden of a great deal of social entertainment whilst on shore, I only hope that in the informal and Bohemian welcome of your medical confrères in this city, you will find something of pleasure to remember after your travels are over, and the glamor of an historic occasion has passed away.

I have also much pleasure in welcoming this evening, the other distinguished visitors who have honored us with their presence, and I sincerely hope they will spend a pleasant evening. Now, Gentlemen, we have a long program which I hope you will all thoroughly enjoy, and, in conclusion I must again express my pleasure at having the privilege of welcoming you here."

After the toasts "The King," "The President U.S.A.," had been submitted by Dr. Inglis and drunk with musical honors, Dr. McDowell proposed the toast "The Fleet Surgeon and Surgeons of the American Fleet."

Dr. McDowell, in proposing the toast of the evening said: "Mr. President, Fleet Surgeon Curtis and Gentlemen, I rise to propose the health of "The Fleet Surgeon, and the Surgeons of the American Fleet." Conscious of the honor of this pleasant duty being placed in my hands on this historic occasion, and proud of being the medium of conveying to our guests the most cordial and enthusiastic fraternal greetings, not only from the members of the Auckland Division, but from the whole of the British Medical Association of New Zealand."

"As an Aucklander by birth, I would like to say at the outset how fully I share in the gratification of our community that the first meeting of the Great White Fleet with the representatives of the British Navy, has taken place in the Waitemata, whose meaning, "sparkling, peaceful water," is surely a happy omen. We have heard a great deal this week of the strong ties which link our two nations together—a common race, language, literature and religion: but the most powerful bond of all is manifested by this company tonight—that of a common calling, a professional brotherhood. We are comrades in arms in the ceaseless warfare against disease, deformity and death. Our minds are bent upon the solutions of the same great problems, and our hands are trained by the same methods to do deftly the work required of them, and our hearts are stirred by the same impulses and aspirations to prevent, to cure, or to assuage the ills of human kind."

"We welcome our guests, in the first place, as distinguished representatives of American medicine, the great names of which we honor, and the splendid achievements of which we gratefully recognise along with them. Let me mention but three names as typical of many esteemed original contributors to the advancement of surgical and medical science since the beginning of last century. Ephriam McDowell, of Kentucky, the "Father of Ovariotomy," James Marion Sims, the celebrated gynecologist and founder of hospitals for women, and Oliver Wendell Holmes, for although as the genial 'Autocrat of the Breakfast Table,' he won fame and love in circles far beyond that of medicine, yet it is well to recall the fact that by his essay on "The Contagiousness of Puerperal Fever," published so far back as 1843, he anticipated by a generation the findings of science in a most important department of pathology. To some of the latter masters of American medicine our president has alluded in his introductory words of welcome, and if you could go the rounds of our private libraries you would find the textbooks of these great authorities in surgery, gynecology and medicine standing cheek by jowl with those of our own British writers, and I am bound to say they would, in many cases, give evidence of more extensive usage. Your current medical literature such as the *Therapeutic Gazette,* that excellent compendium of up-to-date information has a wide circulation amongst us, and the *Journal of the American Medical Association,* and other periodicals are read and valued by us. But, after all, the paramount example of the community of interest in medical affairs throughout Anglo-Saxondom is furnished by the career of Professor William Osler, by birth a native of our sister Dominion of Canada, he made his early reputation at McGill University, Montreal, and then he was called by America to the great Johns Hopkins University, where he firmly established his fame, and finally, as was most fitting, the Motherland claimed him as her own, and honored him with the best post in her gift, that of Regius Professor of Medicine at Oxford."

"We welcome our guests, in the second place, as experts in a particular branch of medicine. This is, as we all recognise, the day of specialism. I was reading the other day—I think it was in one of your own magazines—about a speaker, who was descanting upon this very topic at a medical congress. He had just declared that if the human body was divided into two halves by a line drawn around it at a level of the umbilicus, the upper half would be claimed as the exclusive concern of one group of specialists, and the lower half by another distinct group, when he was interrupted by a voice calling out "you would omit by that division of the body the most important specialists of all, the Naval Surgeons."

Well, gentlemen, I feel sure that in all seriousness we are ready to admit tonight that the Naval Surgeons, especially those of the American Navy, judged by their exploits of the past decade, are indeed, to be classed among the most important of all specialists. They have had an opportunity of demonstrating their quality in warfare, but they have, above all, distinguished themselves in conquests of peace in the realms of public hygiene and tropical medicine, for have they not, in conjunction with their sister military service, practically succeeded in banishing yellow fever, malaria, and other devastating pests from the new possessions of the United States in Cuba, Porto Rico, the Philippines, and Panama?"

"A minor chord in our enjoyment tonight is the thought that at an early hour tomorrow morning we must say farewell to our new-found friends. We have felt greatly honored by your presence among us, we have much enjoyed our brief companionship with you, and we now desire to give you as brotherly a send-off as lies in our power. We trust you will have a real good time, or in your own expressive phrase a "bully" time, and we hope that when you finally bid adieu to Australia, you will carry away the feeling that your "John Bully" time has been the most "bully" of all your "bully" times."

"Last Sunday morning the Union Jack and the Stars and Stripes floated side by side to welcome the Fleet's arrival, but now, at the close of the week, after such happy intimacy, we realise that the officers and men of the American Navy have drawn the flags nearer together, yea have, indeed succeeded in entwining them together into a fair and firm "sailor's knot."

"I now call upon you to charge your glasses and drink to the health of the Surgeons of the Fleet, the pioneers in this good work, and let us also toast to the dawning of the day, when, with a fuller knowledge, and a freer intercourse, the "sailor's knot" will be transformed into a "true lover's knot," a fit symbol of the relations between the two branches of the Anglo-Saxon race, a mighty duality in unity, a power making for liberty, equality and fraternity among the nations of the earth."

Fleet Surgeon Curtis, in reply, related some of his experiences during the visit of the Fleet to the South American ports, and referred to the excellent work which had been done in Rio Janiero by medical experts who had now made it one of the healthiest and cleanest of cities in the world—a contrast to what it was a short time ago—a hotbed of smallpox and yellow fever. Nowhere had they met with a more friendly welcome than in Auckland, and they had been greatly impressed with the beautiful scenery, excellent climate, and general prosperity of the city,"

In a speech replete with characteristic American humor, Surgeon Blackwood replied on behalf of the surgeons of the Fleet. Dr. Bedford then proposed the toast "The Visitors."

A most enjoyable musical program arranged by T. E. Midgely was submitted. Items were contributed by surgeon Traynor (U.S.S. Georgia, Drs. Mason, La Praibe and Guinness, the Lyric Quartette, and Messrs. Manning, Abrahams, Poore and Meeston.

The meeting, which will live long in the memories of all who were present, was marked throughout with enthusiasm and *camaraderie,* and many will look with pleasure to renewing acquaintance with members of the profession as represented in the American Fleet.

